# Inhibition of Growth and Metastasis of Colon Cancer by Delivering 5-Fluorouracil-loaded Pluronic P85 Copolymer Micelles

**DOI:** 10.1038/srep20896

**Published:** 2016-02-11

**Authors:** Pengxi Zhu, Naping Zhao, Dandan Sheng, Jing Hou, Chong Hao, Xue Yang, Bing Zhu, Shanshan Zhang, Zhipeng Han, Lixin Wei, Li Zhang

**Affiliations:** 1 Department of Phamacy, Changhai Hospital, the Second Military Medical University, Shanghai, 200433, China; 2 Tumor Immunology and Gene Therapy Center, Eastern Hepatobiliary Surgery Hospital, the Second Military Medical University, Shanghai, 200433, China

## Abstract

Hepatic metastasis is the leading cause of mortality of colon cancer, which is still lack of an effective therapy. A new delivery system, pluronic P85 block copolymers, conveying chemotherapeutic agent 5-fluorouracil (5-Fu) for inhibiting growth and metastasis of colon cancer was designed and developed. In this study, we demonstrated that 5-Fu produce strong pesticide effect at lower doses in the present of pluronic P85 compared with control groups. The migration and invasion of HCT116 cells and RKO cells were examined and the results showed that migration and invasion capacities of HCT116 cells and RKO cells were reduced by administering 5-Fu/P85 copolymer micelles *in vitro* and *in vivo* which indicating an effectively activity. Interestingly, the content of CD133 + CXCR4+ cells in HCT116 cancer cells and RKO cells treated by 5-Fu/P85 copolymer micelles was decreased. Importantly, the epithelial-mesenchymal transition (EMT) of CD133 + CXCR4+ cells, which was strongly associated with liver metastasis of colon cancer, was also suppressed by giving 5-Fu/P85 copolymer micelles. The results indicated that 5-Fu/P85 copolymer micelles could inhibit the growth and metastasis of colon cancer, which could be attributed to the decrease of the content of CD133 + CXCR4+ cells and suppression of EMT of CD133 + CXCR4+ cells.

Colon cancer is widely spread cancer around the world and currently ranked as the fourth common cause of cancer deaths^1^. The mortality of colon cancer is always ascribed to metastasis and the most prominent type of distant metastasis observed in colon cancer is liver metastasis^2^,^3^. Cancer metastasis is known to be a complex multistep cascade and epithelial-mesenchymal transition (EMT) is regarded as the most important process of tumor invasion and metastasis^4^,^5^. A lot of evidence suggested that the majority of tumors comprise a population of cancer stem cells (CSCs) that are responsible for the maintenance, metastasis and recurrence process of cancer. Recently, a strong association between EMT-associated gene expression and CSCs has been demonstrated^6^,^7^. Our previous study also provided evidence for the existence of a distinct migrating CSC subpopulation of CD133 + CXCR4+ cells in the human colon cancer cells and demonstrated that CD133 + CXCR4+ cancer cells were possible migratory CSC subtypes in colon cancer. EMT is partly involved in these cells acquiring an invasive phenotype and metastatic behavior^8^.

The therapy of colon cancer includes surgical resection, radiotherapy and chemotherapy and so on. However, there is no effective treatment for liver metastasis of colon cancer. 5-fluorouracil (5-Fu) is a chemotherapeutic agents that used in the colon cancer therapy. However, the effect of the treatment is often limited by the toxicity and side-effects^9^,^10^. Pluronic block copolymers were found to be an efficient drug delivery system with multiple effects^11^,^12^,^13^. The chemosensitisation effect of pluronic has been recognized as an effective means of causing an elevation of cancer cell apoptosis by modulating the fluidity of the cell membrane, depleting intracellular ATP^14^, and changing the P-glycoprotein drug efflux pump in multidrug resistant (MDR) cells^15^,^16^,^17^. The study of Daria Y. Alakhova also showed that Doxorubicin/Pluronic SP1049C (a Pluronic-based micellar) effectively suppressing the tumorigEenicity and aggressiveness of P388 cells in a mouse model may be due to enhanced activity of SP1049C against CSC restricting appearance of malignant cancer cell phenotype^18^. In the clinical treatment of colon cancer metastasis, although low dose of 5-Fu produces fewer adverse reactions, it is difficult to achieve an ideal treatment effect. Meanwhile, high doses of 5-Fu therapy that can effectively inhibit the development of colon cancer, which always leads to more adverse reactions and chemotherapy resistance. Therefore we would like to develop a new strategy of 5-Fu therapy, which has the property of effective and fewer side effects. In our study, we considered to combine 5-Fu and pluronic, trying to make 5-Fu produce strong pesticide effect at lower dose.

## Results

### Characterization of 5-Fu/P85 copolymer micelles

**Figure 1:**
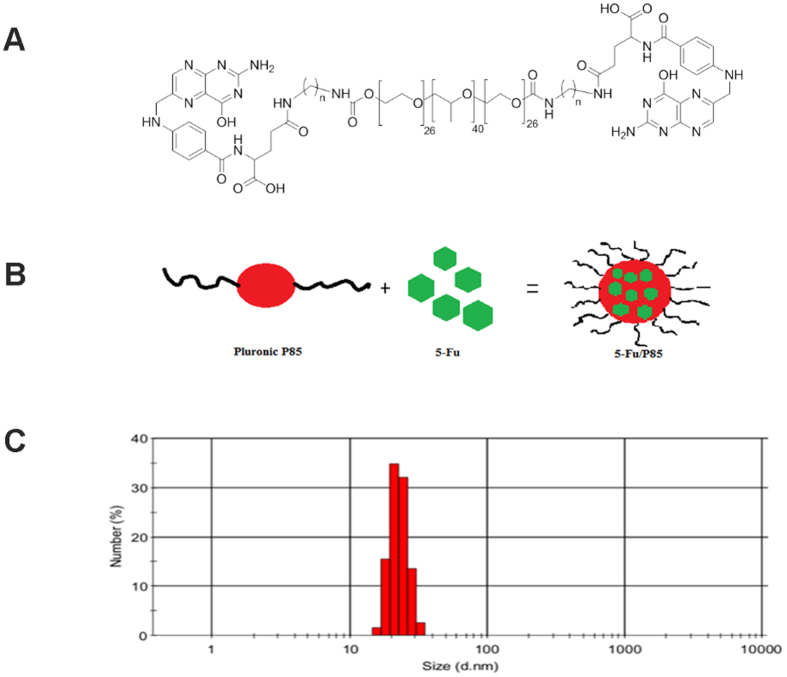
Synthetised 5-Fu/P85 copolymer micelles and the particle size. (**A**) Structural formula of pluronic P85. (**B**) Synthetised 5-fluorouracil and pluronic P85 copolymer micelles by thin-film hydration method. (**C**)The particle size of 5-Fu/P85 copolymer micelles was observed by transmission electron microscopy (TEM) at an acceleration voltage of 80 kV. Digital images were taken with a CCD camera (2048 × 2048 pixels).

Pluronic copolymers consisted of hydrophilic poly (ethylene oxide) (PEO) blocks and hydrophobic poly (propylene oxide) (PPO) blocks and were arranged in triblock structure (PEO-PPO-PEO) (Fig. 1A). Pluronic P85 showed low cytotoxicity and weak immunogenicity in topical and systemic administration. As shown in Fig. 1B,C, P85 could co-load 5-Fu into block copolymers with small particle size (10–100 nm) with +10–35 mV at mass ratio of 2 or higher. The morphology of 5-Fu/P85 copolymer micelles which was shown in supplement Figure 1 was observed by transmission electron microscopy (TEM). The 5-Fu/P85 copolymer micelles characterized by regular spherical structure, uniform size and even distribution. The 5-Fu from nano-micelles was investigated by high-performance liquid chromatography (HPLC). Retention time of 5-Fu was 5.1 min, The HPLC experiments confirmed the assembly of 5-Fu into polymeric micelles.

### 5-Fu/P85 copolymer micelles inhibited the growth and metastasis of colon cancer cells

**Figure 2:**
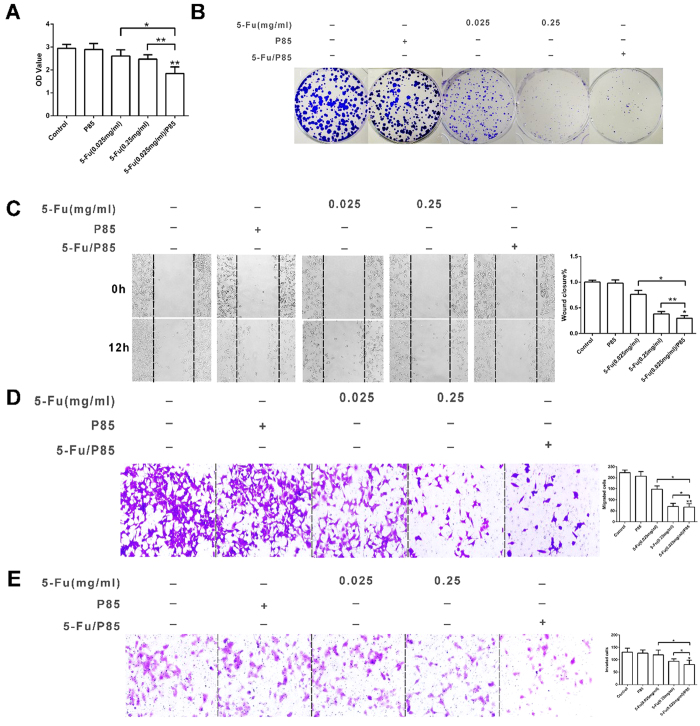
Colon cancer cells HCT116 showed lower proliferative and migratory capacity when treated with 5-Fu/P85 copolymer micelle. (**A**) The cell viability of HCT116 cells treated by different drug groups was measured by cell counting Kit-8(CCK-8). The OD values were detected by microplate reader at 450 nm. (**B**) Clonogenic assay was performed to investigate the clone forming ability of HCT116 cells treated with 5-Fu/P85 copolymer micelle. The cells in six well culture plates were stained by crystal violet. (**C,D**) Cell migration ability was tested by wound-healing assay and transwell migration assay. (**E**) Transwell invasion assay was performed to detect the invasive ability of HCT116 cells. The boyden chambers were stained by crystal violet in transwell migration and invasion assays. The number of cells was counted under a microscope. (Images in (**C**–**E**) were taken by microscope under ×200 magnification. The concentration of 5-Fu was 0.025 mg/ml for 5-Fu/P85 copolymer micelle. *P < 0.05; **P < 0.01.).

The efficacy of 5-Fu/P85 copolymer micelles against HCT116 colon cancer cells and RKO cells were evaluated. HCT116 cells and RKO cells were incubated with P85, 5-Fu (0.025 mg/ml), 5-Fu (0.25 mg/ml) and 5-Fu (0.025 mg/ml)/P85 respectively for 48 h, and then the cell viability was examined by CCK-8 assay. As shown in Fig. 2A and supplement Figure 3A 5-Fu (0.025 mg/ml)/P85 significantly inhibited the cell viability of HCT116 cells and RKO cells compared with control groups. CFU-F assay was employed to observe the effect 5-Fu (0.025 mg/ml)/P85 on the growth of HCT-116 cells. The results showed that colony formation of HCT116 cells incubated with 5-Fu (0.025 mg/ml)/P85 exhibited less CFU-F numbers than control groups (Fig. 2B), which suggested that 5-Fu/P85 copolymer micelles could inhibit the proliferation and clone formation ability of colon cancer cells.

In order to investigate whether 5-Fu/P85 copolymer micelles could inhibit the metastasis of colon cells, we examined the effect of 5-Fu/P85 copolymer micelles on cell motility by wound-healing assay. As shown in Fig. 2C and supplement Figure 3B, HCT116 cells and RKO cells pretreated with 5-Fu/P85 copolymer micelles exhibited significantly attenuated mobility compared with groups. The migration and invasion of HCT116 cells and RKO cells were investigated and the results indicated that 5-Fu/P85 copolymer micelles could effectively inhibit the migration and invasion of HCT116 cells and RKO cells. As shown in Fig. 2D,E, the numbers of migratory and invasive cells treated with 5-Fu/P85 copolymer micelles were significantly less than those in 5-Fu (0.025 mg/ml) group (average 54.7% and 32.5% less respectively, P < 0.01) and slightly less than those in 5-Fu (0.25 mg/ml) group (average 4.3% and 13.8% less respectively, P < 0.05), which was consistent with the results of wound healing assay. Additionally , similar results were found in RKO cells which were shown in supplement Fig. 2C,D.These results indicated that 5-Fu/P85 copolymer micelles shows promising effects in inhibiting the growth and metastasis of CRC.

### 5-Fu/P85 copolymer micelles inhibited the development of colon cancer *in vivo*

**Figure 3:**
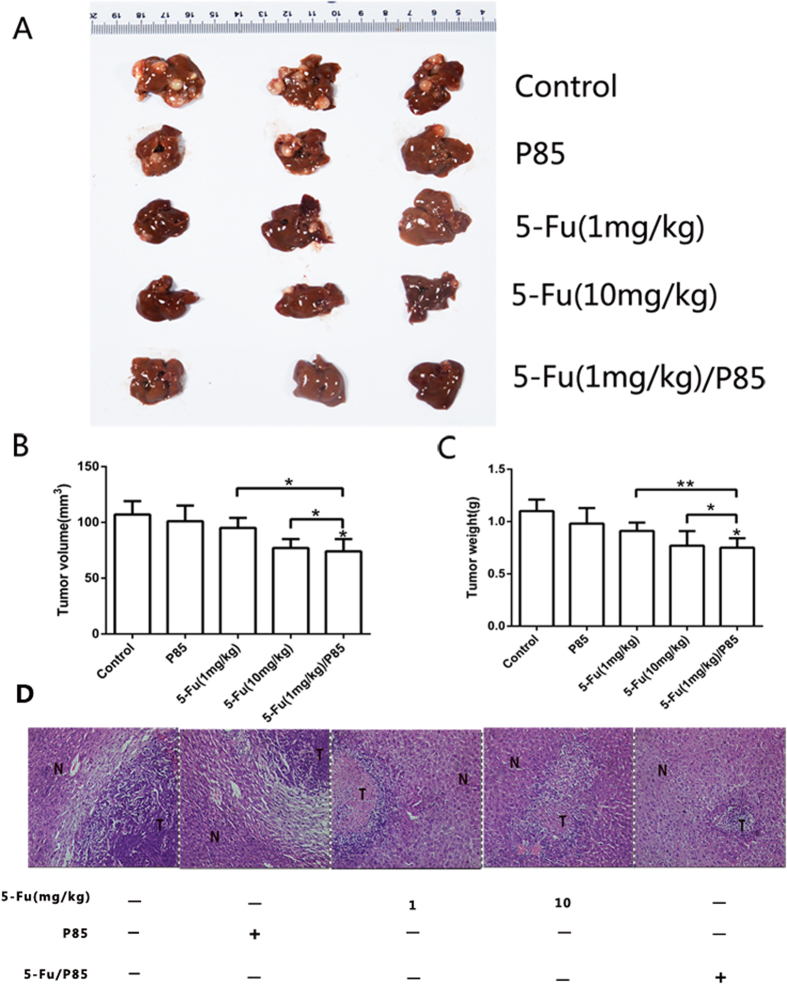
5-Fu/P85 copolymer micelles could inhibit liver metastasis of CRC *in vivo*. **(A**) 5-Fu/P85 copolymer micelles could inhibit metastases in liver by general observation. (**B,C**) Calculation of tumor volume and weight. (**D**)A low frequency of lesions in 5-Fu/P85 group was observed by H&E staining (Optical microscope × 200 magnification, N:normal tissue, T:tumor tissue). (The concentration of 5-Fu was 1 mg/kg for 5-Fu/P85 copolymer micelle. *P < 0.05; **P < 0.01).

To confirm an antitumor effect of 5-Fu/P85 copolymer micelles against liver metastasis of colon cancer, we used nude mouse splenic vein metastasis model. As shown in Fig. 3A, few liver metastatic nodules were observed in 5-Fu (1 mg/kg)/P85 group and most of the liver was free of tumor metastasis. The tumor volume and weight of 5-Fu (1 mg/kg)/P85 group were significantly lower than control groups and 5-Fu (1 mg/kg) group and mildly lower than 5-Fu (10 mg/kg) group (Fig. 3B,C). Moreover, histological analysis demonstrated that metastasis nodules occupied most of the liver when mice were treated by saline and a high frequency of lesions was observed in 5-Fu (10 mg/kg) group. However, histological analyses showed a low frequency of lesions in 5-Fu (1 mg/kg)/P85 group (Fig. 3D).

**Figure 4:**
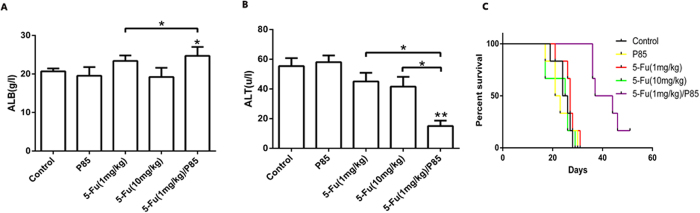
5-Fu/P85 copolymer micelles could improve liver function and prolong survival time of mice. (**A,B**) The liver function indexes ALB and ALT were detected. (**C**) Kaplan-Meier survival analysis was performed to determine the survival of different mice groups (n = 6). (The concentration of 5-Fu was 1 mg/kg for 5-Fu/P85 copolymer micelle. *P < 0.05; **P < 0.01).

As shown in Fig. 4A,B, the mice treated with 5-Fu (1 mg/kg)/P85 had a better liver function compared with control groups. The Kaplan-Meier survival analysis suggested that the survival of 5-Fu (1 mg/kg)/P85 group was also longer than those of the other groups (Fig. 4C). Compared with control and 5-Fu (1 mg/kg) group, the survival rate of 5-Fu (1 mg/kg)/P85 treated group prolonged 40.8% and 36%, respectively. These results suggested that 5-Fu/P85 copolymer micelles could effectively restrict the liver metastasis of colon cancer.

**Figure 5:**
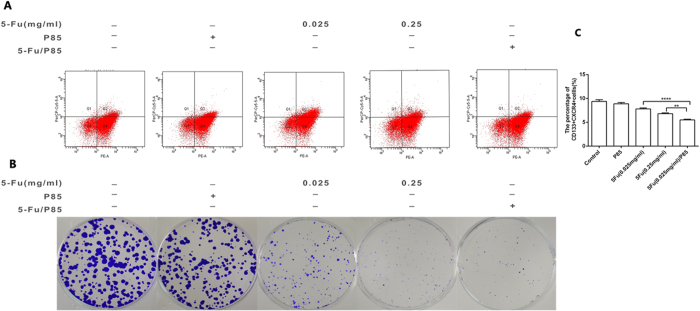
Administration of 5-Fu/P85 copolymer micelles significantly decreased the content of CD133 + CXCR4+ cells in HCT116 cancer cells and inhibited the colony-forming ability of CD133 + CXCR4+ cells. (**A**)The percentage of CD133 + CXCR4+ cells was analyzed by flow cytometry. (**B**) The clonogenic capacity of CD133 + CXCR4+ cells was investigated by CFU assay. The cells in six-well culture plate were stained by crystal violet and the number of colonies containing ≥20 cells was counted under a microscope. (**C**) Quantitative bar graphs showing CD133 + CXCR4+ cell counts determined by flow cytometry. (**P < 0.01;***P < 0.001).

It has been reported that in many cancers there exists a subpopulation of migrating colon cancer which are potentially responsible for cancer metastasis and CXCR4 has been reported to be associated with the cancer cell metastasis phenotype^19^,^20^,^21^. Our previous study indicated that CD133 + CXCR4+ cancer cells are possible migratory CSCs subtypes in colon cancer^8^. So we detected the content of CD133 + CXCR4+ cells in HCT116 cells with different indicated treatments by flow cytometry and the results showed that the content of CD133 + CXCR4+ cells in HCT116 cells pretreated with 5-Fu/P85 copolymer micelles was less than that of HCT116 cells pretreated with 5-Fu or P85 alone group (Fig. 5A,C). As shown in Fig. 5B, the colony formation of CD133 + CXCR4+ cells treated by 5-Fu/P85 copolymer micelles exhibited less CFU-F numbers than control groups, which suggested that 5-Fu/P85 copolymer micelles could inhibit colorectal stem cancer cells proliferation. As shown in supplement Fig. 4A–C, similar results were found in RKO cells. These results indicated that the metastasis-inhibitory effect of 5-Fu/P85 copolymer micelles on HCT116 cells could be ascribed to decreasing the content of CD133 + CXCR4+ cells.

### 5-Fu/P85 copolymer micelles suppressed epithelial-mesenchymal transition of CD133 + CXCR4+ cells

**Figure 6:**
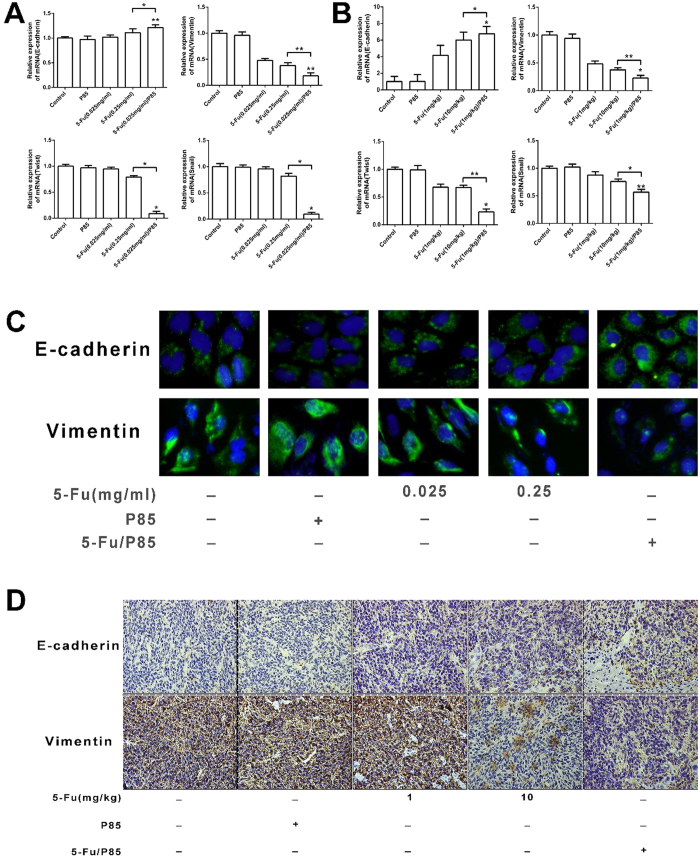
EMT of CD133 + CXCR4+ cells in HCT116 cell line and mice liver cancer tissues was suppressed by delivering 5-Fu/P85 copolymer micelles. (**A**) Relative mRNA expression levels of E-cadherin, vimentin, twist and Snail in CD133 + CXCR4+ cancer cells by qPCR. (**B**) The mRNA expression levels of EMT related genes in mice liver cancer tissues were detected by qPCR. (**C**) Immunofluorescence analysis of E-cadherin and vimentin expression in CD133 + CXCR4+ cancer cells separated from HCT116 by FACS (Fluorescence microscopy ×400 magnification). (**D**) E-cadherin and vimentin expression in liver cancer tissue of mice were tested by immunohistochemistry under optical microscope ×200 magnification. (*P < 0.05; **P < 0.01).

Many studies reported that epithelial-mesenchymal transition (EMT) of CSCs plays a key role in the metastatic activity of human cancers^22^,^23^,^24^,^25^,^26^. We evaluated the mRNA levels of EMT markers and transcription factors including E-cadherin, vimentin, snail and twist in HCT116-derived and RKO-derived CD133 + CXCR4+ cells by qPCR. The expression of E-cadherin was up-regulated in CD133 + CXCR4+ cells pretreated with 5-Fu/P85 copolymer micelles compared with control groups. Vimentin, snail and twist were down-regulated in CD133 + CXCR4+ cells pretreated with 5-Fu/P85 copolymer micelles than control groups (Fig. 6A and supplement Figure 5A). As shown in Fig. 6B, we also detected the mRNA expression of the above EMT-related genes in the mice colon cancer metastatic liver tissues, and the results showed that compared with mice tissues from control groups, the tissue from the group administered with 5-Fu/P85 copolymer micelles had higher E-cadherin expression and lower vimentin, snail and twist expression, which was consistent with the result in Fig. 6A. The results of qPCR were confirmed by immunofluorescent staining in CD133 + CXCR4+ cells with indicated treatment (Fig. 6C and supplement Figure 5B). E-cadherin and vimentin expression in liver metastasis tissue of mice were detected by immunohistochemistry. The E-cadherin expression level was found to be significantly higher and the expression level of vimentin was lower in liver metastasis tissues of mice treated with 5-Fu/P85 copolymer micelles than those in liver metastasis tissues from control groups (Fig. 6D). These results suggested that 5-Fu/P85 copolymer micelles could significantly suppress EMT of CD133 + CXCR4+ cells, which might contribute to their liver metastasis-inhibitory effect.

## Discussion

Colon cancer is one of the most common tumor with high metastatic in past decades. However, colon cancer is not sensitive to chemotherapeutics results in liver metastasis. It is necessary to look for a new resistance mechanism and developing effective chemotherapeutics. Polymer-based drug delivery systems have emerged from the laboratory bench in the 1990’s as a promising therapeutic strategy for the treatment of cancer and other devastating diseases^27^,^28^,^29^,^30^. Pluronic block copolymers with multiple -effects were reported to be an efficient drug delivery system. It can be intake drugs into the core of the micelles and increased sensitivity and reduced drug toxicity and side-effect. Cancer stem cells (CSCs) with high invasive and metastatic originally lead to the maintenance, metastasis and recurrence process of colon cancer. It is crucial to control the CSCs of colon cancer and explore the mechanism behind.

In this work, a new co-delivery system, pluronic P85 block copolymers , conveying chemotherapeutic agent 5-Fu, one of the broad spectrum anticancer agents used in the treatment against colon cancer, gastric cancer, breast cancer, ovarian cancer, bladder cancer and so on^31^, was designed and developed to achieve reinforced drug curative effect. We performed a series of experiments in order to take a further insight into the therapeutic effect of 5-Fu/P85 copolymer micelles on colon cancer. Firstly, the results of cell viability and CFU-F assay showed that 5-Fu/P85 copolymer micelles inhibited the growth of colon cancer cells. And also, the two colon cancer cells pretreated with 5-Fu/P85 copolymer micelles displayed lower capacity of migration and invasion *in vitro*, as compared to their control counterparts. To determine whether 5-Fu/P85 copolymer micelles are effective, tumor development and liver metastasis experiments were carried out *in vivo*. And the results showed that liver metastatic foci in 5-Fu/P85 copolymer micelles pretreated groups were significantly less than those of control groups. And also, the volumes of metastatic tumor exhibited the similar trends. The findings of the above experiments illustrated that 5-Fu/P85 copolymer micelles could inhibit the growth and metastasis of colon cancer *in vitro* and *in vivo*.

There is a group of primitive stem cells from colon cancer, defined as colorectal cancer stem cells, which bears features including the ability to self-renew, differentiate into defined progenies, initiate and sustain tumor growth^32^. These cells are resistant to conventional radiotherapy and chemotherapy. Chemotherapy targets to CSCs may represent a novel therapeutic approach for the treatment of colon cancer^33^,^34^. Colorectal cancer stem cells are enriched in colon tumors following chemotherapy and remain capable of rapidly regenerating tumors from which they originated^35^, resulting in chemoresistance^34^,^36^. However, our data showed that the content of CD133 + CXCR4 + cells in HCT116 cells and RKO cells pretreated with 5-Fu/P85 copolymer micelles was less than those in control groups. This suggested that 5-Fu/P85 copolymer micelles could be a novel therapeutic strategy for CRC.

Mani *et al*.^7^ have found that EMT generates tumor cells with the properties of CSCs. CSCs might promote the invasion and metastasis of tumors by acquiring some properties of the mesenchyma. Meanwhile, the results of our study indicated that CD133 + CXCR4 + cells derived from HCT116 cells pretreated with 5-Fu/P85 copolymer micelles demonstrated up-regulation of E-cadherin, and down-regulation of snail, twist and vimentin. So, the 5-Fu/P85 copolymer micelles suppressed EMT of migratory CSCs in colon cancer according to our results.

In summary, 5-Fu/P85 copolymer micelles could inhibit growth and metastasis of colon cancer. *In vitro*, the cell growth, migration and invasion capacities of CRC cell lines HCT116 and RKO were reduced by administering 5-Fu/P85 copolymer micelles. *In vivo* antitumor efficacy showed that 5-Fu/P85 copolymer micelles could not only prolong survival rate effectively, but also restrict the liver metastasis in mice model. Further investigations providing one explanation for the inhibitory effect of 5-Fu/P85 copolymer micelles on colon cancer, showed that 5-Fu/P85 copolymer micelles could decrease the content of CD133 + CXCR4+ cancer cells in HCT116 and RKO cell lines and suppress EMT of CD133 + CXCR4+ cancer cells. Therefore, 5-Fu/P85 copolymer micelles could be expected to be a promising new approach for treatment of liver metastasis of CRC.

We elucidated that pluronic P85 conveying chemotherapeutic agent 5-fluorouracil dramatically suppressed the capacity of metastasis and decreased quantity of CSCs in colon cancer. 5-Fu/P85 copolymer micelles could inhibit epithelial-mesenchymal transition in CSCs indicate that it might be a new strategy to reverse chemotherapy resistance. 5-Fu/P85 copolymer micelle could be a promising chemotherapeutic drug on colon cancer therapy.

## Methods

### Materials

Pluronic P85 was purchased from BASF Ltd. (Parispany, NJ, USA), 5-fluorouracil purchased from Bangcheng Pharmaceutical Co., Ltd. (Shanghai, China). McCoy’s5A medium, DMEM medium, fetal bovine serum (FBS), penicillin, streptomycin sulfate, glutamine, and 0.05% trypsin/0.02% ethylenediamine tetraacetic acid (EDTA) solution were purchased from Invitrogen (Carlsbad, CA, USA). Rabbit anti human E-cadherin and Vimentin antibodies were obtained from Thermo (Fremont, CA, USA). Goat anti-human Snail and Twist antibodies were purchased from R&D (Minneapolis, MN, USA). Methanol for HPLC was bought from CNW (Shanghai ANPEL INC. China).

### Preparation of 5-Fu/P85 copolymer micelles by thin-film hydration method

P85 (50 mg) was dissolved in 10 mL acetonitrile contained with 5-Fu (2 mg) in a round-bottom flask. The solvent was evaporated by rotary evaporation at 50 °C for about 1 h to obtain a solid 5-Fu/copolymer matrix. Residual acetonitrile remaining in thin-film was removed under vacuum overnight at room temperature. Then, the resultant film was hydrated with distilled water.

### High performance liquid chromatography (HPLC) identification of 5-Fu

With certain amount of P85and 5FU/P85, after equilibrating at room temperature for 12 h, drug-loaded micelles 0.4 mL were transferred into dialysis membrane (Cellu Sep H1, MWCO ~ 1000), and stirred with the rate of 200 rpm in 1000 mL ddH_2_O at room temperature for 0.5 h. Non-dialyzed Empty micelles, dialysate, dialyzed Empty micelles and drug-loaded micelles (0.1 mL) were sampled and mixed with methanol to depolymerize the micelles and release entrapped 5-Fu. New prepared 5-Fu (500 ng/mL) and the other mentioned four samples were centrifuged (13000 rpm, 5 min) and identified for 5-Fu by using Shmadzu HPLC instrumentation, which was equipped with LC-20AD pumps, DGU-20A3 degasser, SIL-20AC autosampler (5 μL), CTO-20A column oven (35 °C), SPD-20A UV detector (265 nm), Athena C18, 120A column (4.6 × 250 mm, 5 μm, CNW, Shanghai ANPEL INC. China), Athena C18 Guard Cartridge Kit, 120A, (4.0 × 20 mm, 5 μm, CNW, Shanghai ANPEL INC. China). Mobile phrase was a mixture of methanol: water (7:93, v/v), eluted at a flow rate of 1.0 mL/min.

### Cell culture

The human colorectal cancer cell lines, HCT116 and RKO, were cultured respectively with McCoy’s5A Medium and DMEM Medium (GIBCO, Invitrogen, Carlsbad, CA, USA) supplemented with 10% fetal bovine serum (FBS; GIBCO, Invitrogen), 100 units/ml penicillin and 100 mg/ml streptomycin in a humidified incubator under 95% air and 5% CO_2_ at 37 °C.

### Cell counting kit-8 (CCK-8) assay

The measurement of viable cell mass was performed by CCK8 (Dojin Laboratories, Kumamoto, Japan). Cells (5 × 10^3^ cells/well) were firstly seeded in 96-well flat-bottomed plates for 24 h, and then were given different treatment for 48 h. As soon as the treatment was completed, 10 μl solution from CCK-8 was added to each well. These plates were continuously incubated for 1 h in a humidified CO_2_ incubator at 37 °C. Finally, the absorbance of sample taken from each well was measured on a microplate reader (Synergy HT, Bio-Tek) at 450 nm, on the basis of which the percentage of surviving cells of each treated group to the untreated one was plotted.

### Colony formation unit assay

About 1 × 10^3^cells were added into each well of a six well culture plate (three wells for each group). After incubation at 37 °C for 14 days, the cells were washed twice with PBS and stained with 0.1% crystal violet solution. The number of colonies containing ≥20 cells was counted under a microscope.

### Wound healing assay

For migration assay, wound-healing assay was done. Cells (5 × 10^5^) were seeded on 6-well dish and incubated for 24 h, monolayer was then disrupted with a cell scraper (1.2 mm width), and photographs were taken at 0 and 12 h in a phase-contrast microscope. Experiments were carried out in triplicate, and four fields of each point were recorded.

### Transwell assay

For transwell migration assays, 2 × 10^4^ cells were plated in the top chamber with the non-coated membrane (24-well insert, pore size: 8 mm). For invasion assays, 5 × 10^4 ^cells were plated in the top chamber with Matrigel-coated membrane (24-well insert, pore size: 8 mm). Cells in medium without serum were plated in the upper chamber, and the medium containing 5% FBS was added in the lower chamber as a chemoattractant. After 24 h of incubation at 37 °C, the cells were fixed in 4% formaldehyde and stained with crystal violet dye, and the cells that invaded through the pores to the lower surface of the filter were counted under a microscope. Three invasion chambers were used per condition. The values obtained were calculated by averaging the total number of cells from three filters.

### Animal model

All procedures involving animals were performed in accordance with the institutional animal welfare guidelines of Second Military Medical University and approved by the Ethics Committee of Changhai Hospital. 8-week-old male nude mice were randomly assigned to experimental and control groups (n = 10). HCT116 cells were injected into the splenic vein of mice at 2 × 10^6 ^cells/injection site. After 4 days, mice was injected through tail vein with saline; P85; 5-Fu (1 mg/kg); 5-Fu (10 mg/kg) and 5-Fu (1 mg/kg)/P85 copolymer micelles. Treatments were administered every day for 14 consecutive days, part of them were sacrificed. Tumor weight and volume were measured; the liver tissue was immediately excised, imaged for H&E staining and immunohistochemistry staining. Another part of mice were observed until death.

### Measurement of liver injury

Blood samples were collected from mice after treated by drug for 14 days. The plasma albumin (ALB) and alanine aminotransferase (ALT) levels were tested with a biochemical autoanalyzer (Fuji Medical System, Tokyo, Japan) according to the manufacturer’s instructions.

### Flow cytometry analysis

The CD133 + CXCR4+ cancer cell content was determined by flow cytometry. Suspensions of colon cancer cells (10^7^/ml) were sorted according to the expression of CD133 and CXCR4 with fluorescence activated cell sorting system (FACS, Becton Dickinson, San Jose, CA, USA) following multicolor staining as described for flow cytometric analyses. Separated subpopulations were reanalyzed for purity and then used in subsequent experiments

### Real-Time PCR

The cells and liver metastasis were collected to extract total mRNA with Trizol reagent (Invitrogen, Carlsbad, CA, USA). Expression of mRNA was determined by qPCR using SYBR Green Master Mix (Applied Biosystems, Foster City, CA, USA). Total sample RNA was normalized to endogenous GADPH mRNA. The sequences of primers used in this study are shown in Table 1. Thermal cycling conditions included an initial hold period at 95 °C for four minutes; this was followed by a two-step PCR program of 95 °C for 20 seconds and 72 °C for 30 seconds repeated for 40 cycles on Mx4000 system (Stratagene, La Jolla, CA, USA).

### Immunofluorescence staining

About 10^5^ cells were seeded on a 24-well dish. After 24 h, the cells were washed with phosphate-buffered saline (PBS) twice and fixed in 4% paraformaldehyde and 0.1% Triton X 100 in PBS buffer at 4 °C for 30 minutes. After being washed with PBS, the cells were incubated with the blocking solution (10% goat serum in PBS), and then incubated with the primary antibodies overnight, washed with PBS, and finally incubated with secondary antibodies (Invitrogen) at 37 °C for 2 h. After stained with DAPI, all matched samples were photographed using immunofluorescence microscope and identical exposure times.

### Immunohistochemistry staining

Sections of tumor tissue were cut from the specimens for immunohistochemistry analysis. The samples were fixed in acetone, air-dried, and subsequently bathed in TBS solution (pH 7.6). The endogenous peroxidase activity was blocked with 3% hydrogen peroxide. For E-cadherin or Vimentin immunohistochemistry staining, a goat polyclonal antibody (Santa Cruz Biotechnology, Inc., Santa Cruz, CA) was used at the dilution of 1:100 and incubated at 4 °C overnight. After reacting with a biotinylated secondary antibody for 2 h, antigen-antibody reactions were visualized using streptavidin-horseradish peroxidase conjugate (DAKO LSAB kit; DAKO, Los Angeles, CA), with 3-amino-9-ethylcarbazole as the chromogen. All slides were counterstained with hematoxylin.

### Statistical Analysis

All data, expressed as means ± SEM, were from at least three separate experiments. Groups were compared by t-test. P < 0.05 was considered as significant.

## Additional Information

**How to cite this article**: Zhu, P. *et al*. Inhibition of Growth and Metastasis of Colon Cancer by Delivering 5-Fluorouracil-loaded Pluronic P85 Copolymer Micelles. *Sci. Rep.* **6**, 20896; doi: 10.1038/srep20896 (2016).

## Supplementary Material

Supplementary Information

## Figures and Tables

**Table 1:** List of primers used for q PCR (GAPDH was used as an internal control).

	GeneProduct	Primers Product
MOUSE	E-Cadherin	Fw: 5′- CAAAGACAGAGGAGAGAAAAGGAGA -3′
		Rv: 5′- GCCAGAAAACATTGGTTGAGATAAG -3′
	Vimentin	Fw: 5′- TGCCTCTGCCAACCTTTTCT-3′
		Rv: 5′- CATCTCTGGTCTCAACCGTCTTAAT -3′
	Twist	Fw: 5′- GAGATGATGCAGGACGTGTCCA -3′
		Rv: 5′- GACTGCTGCGTCTCTTGCGA -3′
	Snail	Fw: 5′- CTGGTTCCTGCTTGGCTCTCTT -3′
		Rv: 5′- GTGGGTTGGCTTTAGTTCTATGGC -3′
	β-actin	Fw: 5′- TGCTGACAGGATGCAGAAGGAG -3′
		Rv: 5′- GTGGACAGTGAGGCCAGGATAG -3′
HUMAN	E-Cadherin	Fw: 5′- TGAAGGTGACAGAGCCTCTGGA -3′
		Rv: 5′- TGGGTGAATTCGGGCTTGTT -3′
	Vimentin	Fw: 5′- TGGCCGACGCCATCAACACC -3′
		Rv: 5′- CACCTCGACGCGGGCTTTGT -3′
	Twist	Fw: 5′- GCCAGGTACATCGACTTCCTCT -3′
		Rv: 5′- TCCATCCTCCAGACCGAGAAGG -3′
	Snail	Fw: 5′- CCTCCCTGTCAGATGAGGAC -3′
		Rv: 5′- CCAGGCTGAGGTATTCCTTG -3′
	DAPDH	Fw: 5′- TGCCAAATATGATGACATCAAGAA -3′
		Rv: 5′- GGAGTGGGTGTCGCTGTTG -3′
