# Supplementary material for: Inhibition of Growth and Metastasis of Colon Cancer by Delivering 5-Fluorouracil-loaded Pluronic P85 Copolymer Micelles
Source: Sci Rep. 2016 Feb 11;6:20896. doi: 10.1038/srep20896 (PMC4750032; doi:10.1038/srep20896)
Supplement: Supplementary Information [file srep20896-s1.pdf]

Inhibition of Growth and Metastasis of Colon Cancer by Delivering 5-Fluorouracil-loaded Pluronic P85 Copolymer Micelles

Pengxi Zhu[1,+], Naping Zhao[1,+], Dandan Sheng[1,+], Jing Hou[1], Chong Hao[2], Xue Yang[2], Bing Zhu[1], Shanshan Zhang[2] & Zhipeng Han[2] ,Lixin Wei[2,*] Li Zhang[1,*],

[1] Department of Phamacy, Changhai Hospital, the Second Military Medical University, Shanghai, 200433, China

[2] Tumor Immunology and Gene Therapy Center, Eastern Hepatobiliary Surgery Hospital, the Second Military Medical University, Shanghai, 200433, China

[+]These authors contributed equally to this work

* Corresponding Author: weilixin_smmu@163.com chyyyckzl@163.com

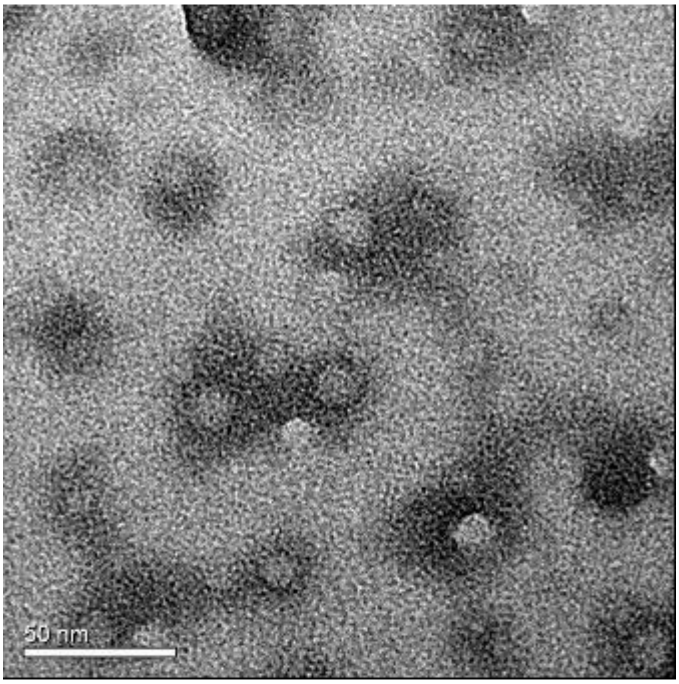

Supplement Figure 1. The morphology of 5-Fu/P85 copolymer micelles was observed by transmission electron microscopy (TEM)

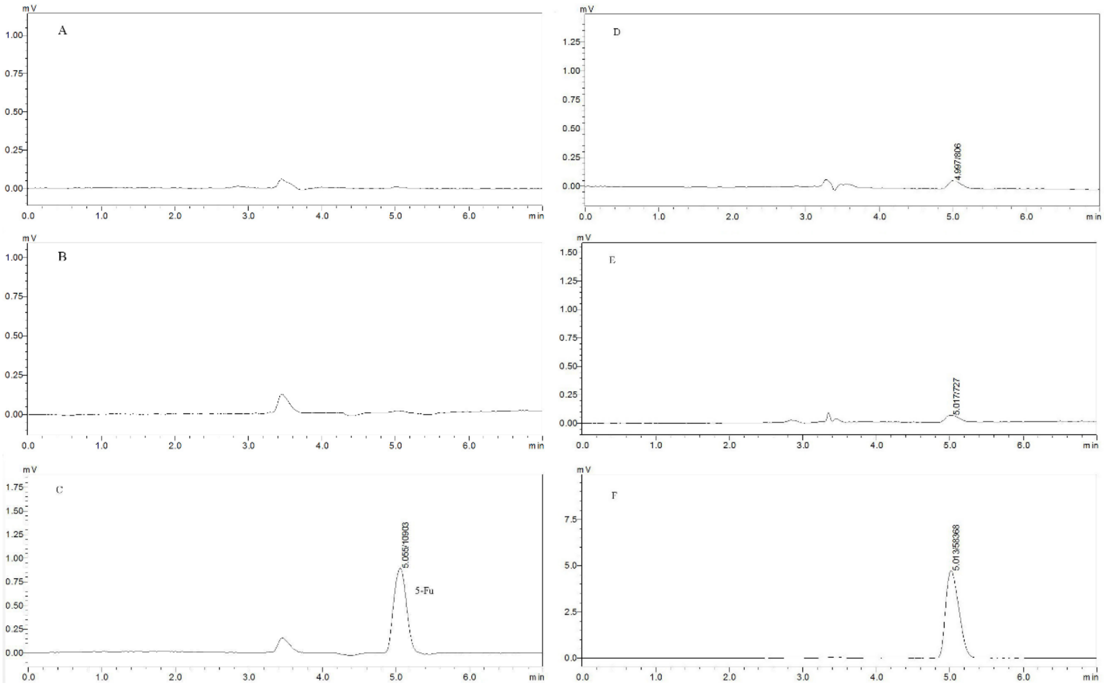

Supplement Figure 2. High performance liquid chromatography (HPLC) identification of 5-Fu. Typical chromatograms of：(A) DDH_2O (B) Empty micelles (C) new prepared 5-Fu (2500 ng/mL); (D) dialyzate (E) dialysed Empty micelles (F) dialysed drug-loaded micelles. The retention time of 5-Fu under the conditions in this study was 5.0 min. All the samples were mixed with equal amount of methanol and prepared under the same conditions.

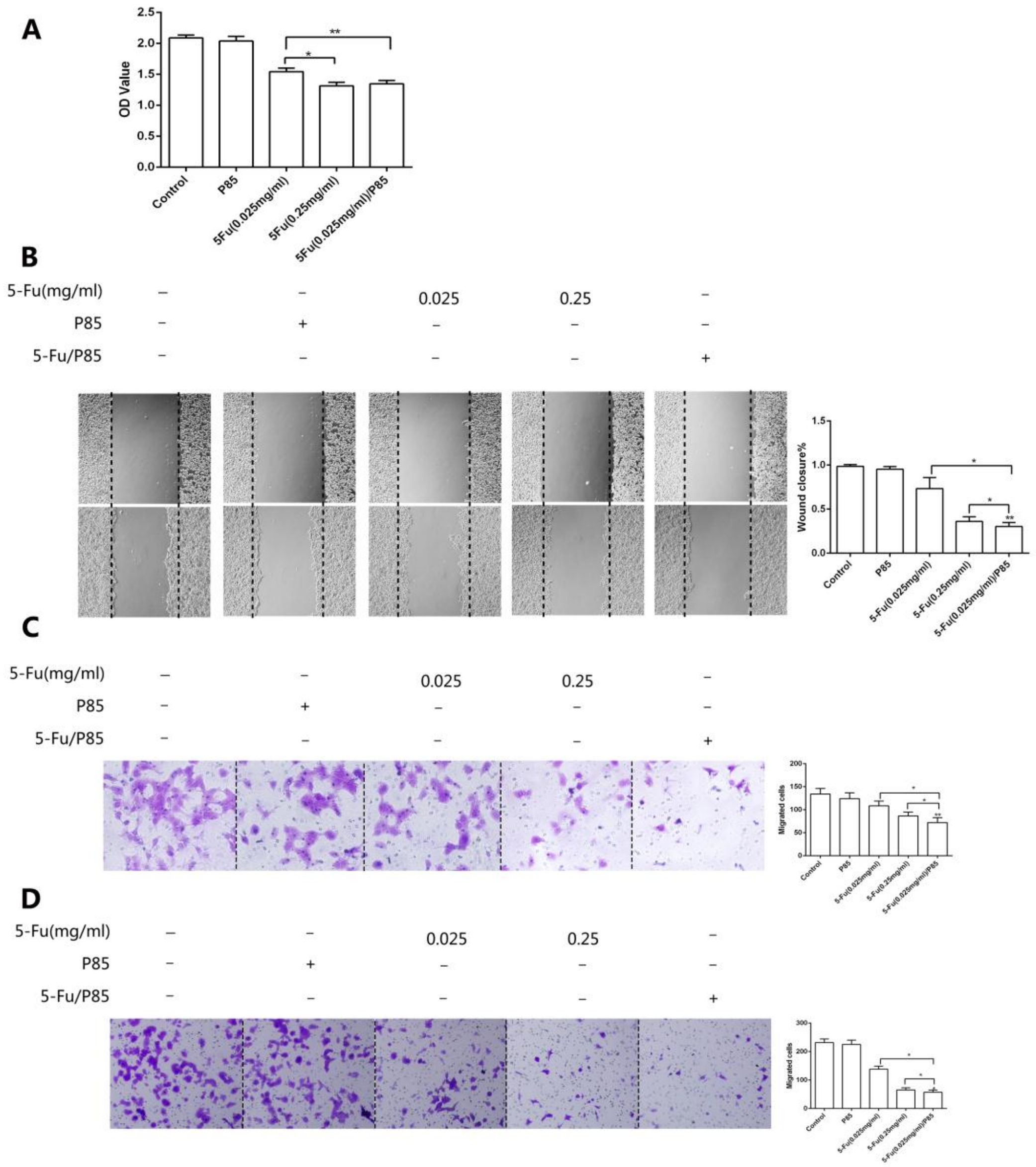

Supplement Figure3. Colon cancer cells RKO showed lower migratory capacity when treated with 5-Fu/P85 copolymer micelle.

(A) The cell viability of RKO cells treated by different drug groups was measured by cell counting Kit-8(CCK-8). The OD values were detected by microplate reader at 450nm. (**B, C**) Cell migration ability was tested by wound-healing assay and transwell migration assay. (**D**) Transwell invasion assay was performed to detect the invasive ability of RKO cells. The boyden chambers were stained by crystal

violet in transwell migration and invasion assays. The number of cells was counted under a microscope. (Images in (**B**), (**C**) and (**D**) were taken by microscope under ×200 magnification. The concentration of 5-Fu was 0.025mg/ml for 5-Fu/P85 copolymer micelle. *P<0.05;*** P<0.001.)

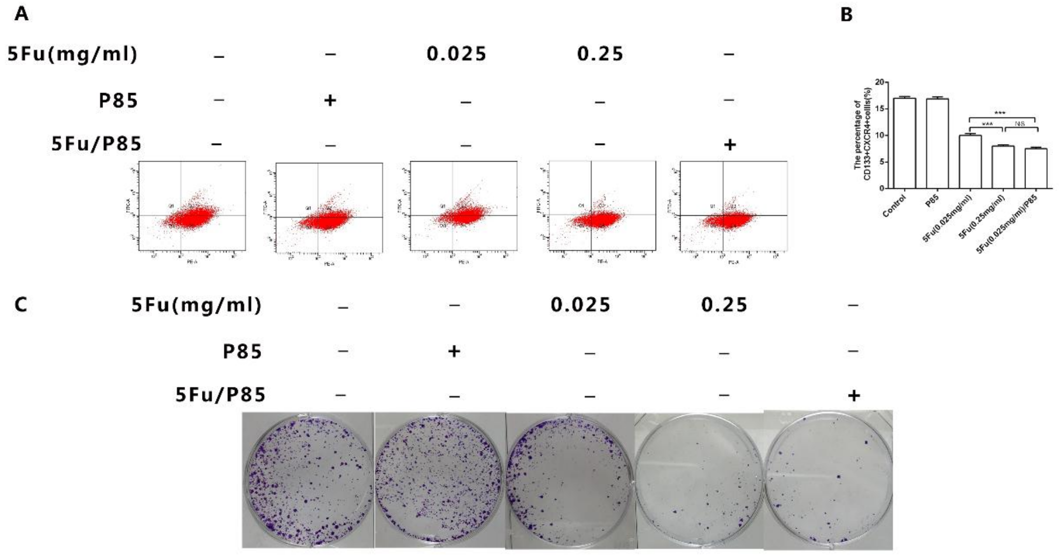

Supplement Figure4. Administration of 5-Fu/P85 copolymer micelles significantly decreased the content of CD133+CXCR4+ cells in RKO cancer cells and inhibited the colony-forming ability of CD133+CXCR4+ cells.

(A)The percentage of CD133+CXCR4+ cells was analyzed by flow cytometry. (B) Quantitative bar graphs showing CD133+CXCR4+ cell counts determined by flow cytometry. (C) The clonogenic capacity of CD133+CXCR4+ cells was investigated by CFU assay. The cells in six-well culture plate were stained by crystal violet and the number of colonies containing ≥20 cells was counted under a microscope. (*** P<0.001)

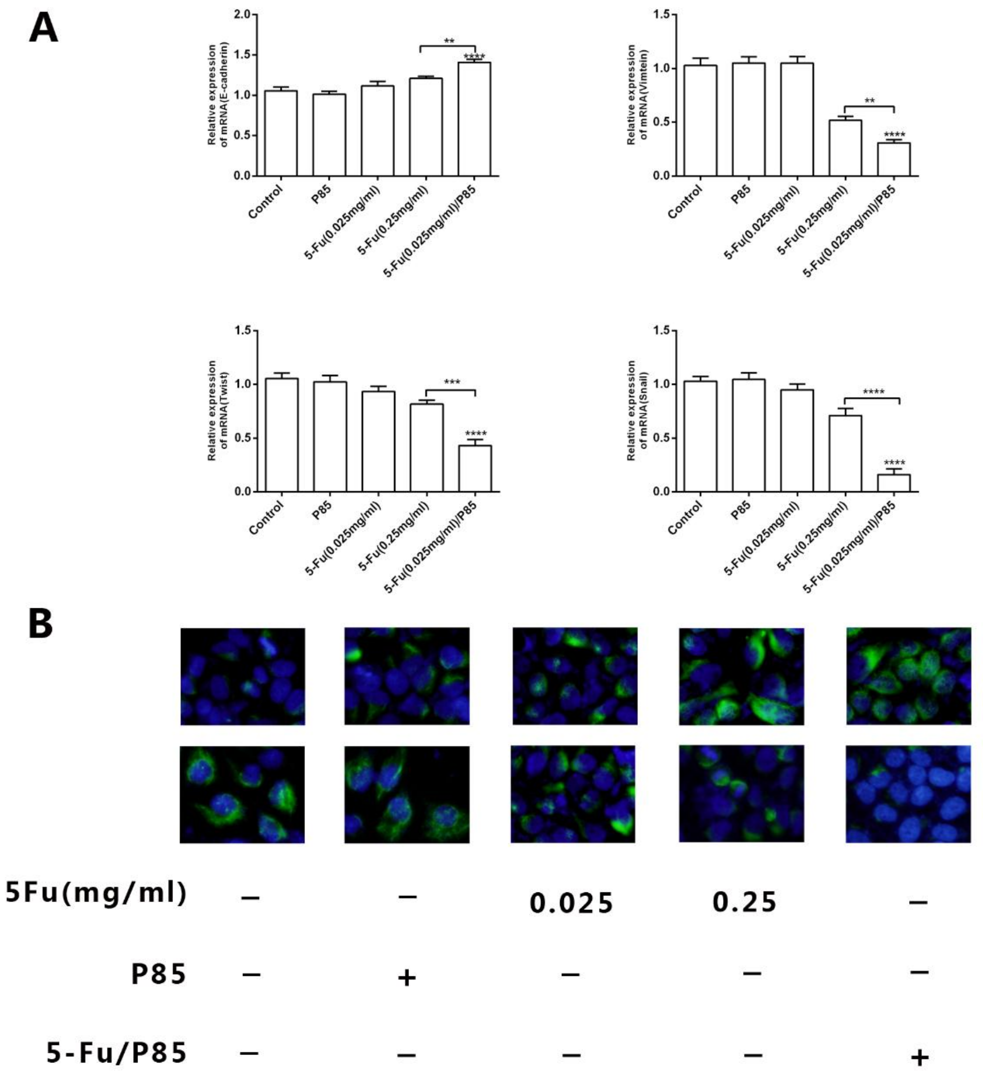

Supplement Figure 5. EMT of CD133+CXCR4+ cells in RKO cell line was suppressed by delivering 5-Fu/P85 copolymer micelles.

(**A**) Relative mRNA expression levels of E-cadherin, Vimentin, Twist and Snail in CD133+CXCR4+ cancer cells by qPCR. (**B**) Immunofluorescence analysis of E-cadherin and vimentin expression in CD133+CXCR4+ cancer cells separated from RKO by FACS (Fluorescence microscopy ×400 magnification). (***P*<0.01; *****P*<0.0001)
